# Characterization, Expression Profiling, and Functional Analysis of *PtDef*, a Defensin-Encoding Gene From *Populus trichocarpa*

**DOI:** 10.3389/fmicb.2020.00106

**Published:** 2020-02-07

**Authors:** Hui Wei, Ali Movahedi, Chen Xu, Weibo Sun, Pu Wang, Dawei Li, Tongming Yin, Qiang Zhuge

**Affiliations:** ^1^Key Laboratory of Forest Genetics & Biotechnology of Ministry of Education, Co-Innovation Center for Sustainable Forestry in Southern China, College of Biology and Environment, Nanjing Forestry University, Nanjing, China; ^2^Jiangsu Provincial Key Construction Laboratory of Special Biomass Resource Utilization, Nanjing Xiaozhuang University, Nanjing, China

**Keywords:** JA, *Populus trichocarpa*, *PtDef*, defensin, Nanlin895 poplar, SA

## Abstract

PtDef cloned from *Populus trichocarpa* contained eight cysteine domains specific to defensins. Quantitative reverse-transcription polymerase chain reaction (qRT-PCR) analysis showed that *PtDef* was expressed in all tissues tested, with lower expression in leaves and higher expression in petioles, stems, and roots. Purified fused PtDef inhibited *Aspergillus niger*, *Alternaria Nees*, *Mucor corymbifer*, *Marssonina populi*, *Rhizopus* sp., and *Neurospora crassa*. PtDef also inhibited the growth of *Escherichia coli* by triggering autolysis. *PtDef* overexpression in Nanlin895 poplar (*Populus* × *euramericana* cv. Nanlin895) enhanced the level of resistance to *Septotinia populiperda.* qRT-PCR analysis also showed that the expression of 13 genes related to salicylic acid (SA) and jasmonic acid (JA) signal transduction differed between transgenic and wild-type (WT) poplars before and after inoculation, and that *PR1-1* (12–72 h), *NPR1-2*, *TGA1*, and *MYC2-1* expression was higher in transgenic poplars than in WT. During the hypersensitivity response (HR), large amounts of H_2_O_2_ were produced by the poplar lines, particularly 12–24 h after inoculation; the rate and magnitude of the H_2_O_2_ concentration increase were greater in transgenic lines than in WT. Overall, our findings suggest that *PtDef*, a defensin-encoding gene of *P. trichocarpa*, could be used for genetic engineering of woody plants for enhanced disease resistance.

## Introduction

Plant defensins are antibacterial peptides that play an important role in plant immunity; they are the primary barrier to invasion by pathogenic bacteria (Ganz, 2003; Zhao et al., 2011). Animal and plant defensins evolved separately and have very different amino acid sequences and cystine connectivity (Shafee et al., 2016). Genes encoding plant defensins can be divided into those with higher and lower sequence homology; these two categories of defensins may have different biological activities (Stotz et al., 2009). Defensin-encoding genes have been identified in Gramineae (Bloch and Richardson, 1991), Cruciferae (Broekaert et al., 1995), gymnosperms (Brodelius and Xue, 1997; Do et al., 2004), and Cucurbitaceae (Da-Hui et al., 2007). Plant defensins are cysteine-rich cationic polypeptides of 45–50 amino acid residues (∼5 kDa) and have antimicrobial activity (Vriens et al., 2014). Defensins have a tertiary structure comprising an αβ (CSαβ) motif consisting of an α-helix and three reverse parallel β-pleated sheets fixed by four disulfide bonds formed by eight cysteines (Yount and Yeaman, 2004). The expression of the genes encoding plant defensins differs among tissues and upon pathogen invasion (De Coninck et al., 2015; Pothana et al., 2019). For example, *Arabidopsis* PDF1.2 is a defense marker gene related to the jasmonic acid (JA) pathway and is upregulated by pathogens. Most plant defensins have antifungal activity (De Coninck et al., 2017), presumably mediated by interaction with specific sphingolipids on the fungal membrane (Thevissen et al., 2004; Cools et al., 2017). Phyto-defensins inhibit protein synthesis (Méndez et al., 1996) and have alpha-amylase and protease activity (Pelegrini et al., 2008). Some plant defensins, but not all, bind to sphingolipids. Plant defensin activity is controlled by many different mechanisms (Parisi et al., 2018). Due to the functional diversity of plant defensin genes, they are widely used for genetic engineering. Transformation of an alfalfa defensin gene into potato enhanced potato resistance to *Verticillium dahliae* (Gao et al., 2001). Transformation of a radish defensin gene into tobacco improved tobacco resistance to *Alternaria longicornis* (Terras et al., 1993), and pea defensins inhibit pathogens and fungi in pea clip epidermis and vascular bundles (Almeida et al., 2002).

Plants defend against diseases using constitutive and induced mechanisms. Induced defense mechanisms play an important role in plant self-protection (Scheres and van der Putten, 2017). The hypersensitivity response (HR) is a type of cell death that evolved to suppress the replication of pathogens and mediates the resistance to plant disease (Fonseca and Mysore, 2019; Seifi and Shelp, 2019). Following pathogen invasion, the HR isolates the pathogen from the host plant, thus suppressing growth of the former (Gabriel and Rolfe, 1990). Cell death caused by the HR stimulates a defense response in adjacent tissues, leading to acquisition of systemic resistance. Therefore, the plant HR involves both cell death and the expression of resistance genes (Fonseca and Mysore, 2019; Seifi and Shelp, 2019). In the early stage of the HR, a large quantity of reactive oxygen species (ROS) is produced by plants, which further activates the HR (Fonseca and Mysore, 2019). The production of ROS, particularly hydroxyl radicals and H_2_O_2_, is an early cellular response to pathogens (Smirnoff and Arnaud, 2019), however, H_2_O_2_ can kill plant cells at high concentrations (Siddique et al., 2014). Antioxidant enzymes – peroxidase dismutase (SOD), peroxidase (POD), catalase (CAT), ascorbic acid peroxidase (APX), glutathione peroxidase (GPX), and glutathione-S-transferase (GST) – protect plants against damage by removing ROS. Phenylalanine ammonia lyase, chalcone synthase, and peroxidase are key enzymes in the biosynthesis of phenolic compounds and in lignin metabolism in plants (Vanholme et al., 2019). Most phenolics directly kill pathogenic bacteria, and lignin enhances the mechanical barrier of the plant cell wall. Upon development of disease, plants activate salicylic acid (SA), JA, ethylene (ET), and abscisic acid (ABA) signaling pathways, which mediate the response of plants to disease (Halitschke et al., 2004; Yang et al., 2018; Islam et al., 2019).

In the presence of disease, plants activate the expression of defense genes by regulating the synthesis of a variety of hormones. The transcription inhibitor, COI1 (coronatine insensitive 1)/JAZ (Jasmonate ZIM-domain), is important for the induction of plant disease resistance by JA and its derivatives. Jasmonoyl-L-isoleucine (JA-Ile) conjugates of JA and isoleucine are produced by plants in a diseased state. JAZ is degraded, which lifts the inhibition of transcription factor activity and induces the expression of JA signaling genes. MYC2 is an important transcription factor in this process, and links JAZ degradation to expression of genes related to JA signaling (Yan and Xie, 2015). SA induces the expression of pathogenesis-related protein (*PR*), resulting in initiation of the HR. SA promotes the acquisition of resistance by accumulation of PR protein and ROS, and can be transported in plants (Cui et al., 2017; Svetaz et al., 2017). The non-expresser of physically related protein 1 (*NPR1*) is the dominant regulator of plant disease resistance. Maintenance of a low SA concentration in plant cells promotes accumulation of NPR1 and activation of TGA (WRKYS and TGACG sequence-specific binding proteins) transcription factors, which induces the expression of genes related to disease resistance (Fu et al., 2012). Previous studies have shown that TGA2, TGA5, and TGA6 inhibit the transcript level of *PR1* in the absence of SA, whereas TGA2, TGA5, and TGA6 are needed to induce PR1 in the presence of SA. In the absence of SA, TGA2 binds to the PR1 promoter, thereby inhibiting its transcription. The protein NIMIN1 can form ternary complexes with TGA2 through interaction with NPR1, at least in yeast. Transcriptional inhibition of TGA2 may be achieved by the interaction of NIMIN1 with a transcriptional co-repressor, without an upper body. It is conceivable that SA allows NPR1 to form different complexes with TGA2 and other TGA factors, such as TGA3, to activate PR1 transcription. The NIMIN1–NPR1–TGA2 complex dissociates in the presence of SA in yeast. Therefore, SA can separate NIMIN1 from the NPR1–TGA transcriptional complex, which may be helpful in activating the NPR1–TGA transcriptional complex (Seyfferth and Tsuda, 2014; Wang et al., 2015). JA and SA exert both synergistic and antagonistic effects on plant disease resistance (Yasuda et al., 2008).

The Poplar is among the most widely distributed and adaptable tree species. It is mainly distributed in temperate and cold-temperate regions of the northern hemisphere (latitude 22–70°N), including China, Russia, Canada, United States, Italy, and France. The Poplar has been used in studies of the genome of perennial plants due to the availability of its genome sequence (Tuskan et al., 2006). Genetic engineering can be applied to improve poplar. *Septotinia populerda* was first discovered as a poplar disease in 1950 (Waterman and Cash, 1950). Following infection by *S. populerda*, brown spots form in infected leaf parts, gradually becoming gray-white holes. If the poplars are damaged year after year, the tree will weaken, enhancing conditions for disease occurrence *via* weak pathogens. Poplar is an important industrial plantation species worldwide; infection by *S. populerda* seriously affects poplar production, in turn significantly reducing the economic and ecological benefits of poplar (Kaul et al., 2010; Wingfield et al., 2015). Plant defensins have antimicrobial activity and play an important role in the stress response, so they are a focus in the research of plant diseases. In this study, we cloned the *PtDef* defensin-encoding gene from poplar. We investigated the tissue-specificity of, and the effect of a variety of stresses on, the expression of *PtDef* by quantitative RT-PCR. Also, PtDef was produced in a prokaryotic system, purified, and its activity evaluated *in vitro*. PtDef induced autolysis of *Escherichia coli* BL21 (DE3) and activated expression of a two-component system (TCS). Moreover, Nanlin 895 poplar (*Populus* × *euramericana* cv. Nanlin 895) was transformed with PtDef by the Agrobacterium method. The transgenic poplar exhibited greater resistance to *S*. *populiperda* than the wild-type (WT) poplar. Furthermore, the expression of *PR1*, *NPR1*, and *TGA* (related to SA signaling) and of *MYC2*, *JAZ*, and *COI1* (related to JA signaling) was evaluated in transgenic and WT poplars infected with *S*. *populiperda*. We explored the plant-hormone signal transduction mechanisms in poplar that mediate the resistance to *S*. *populiperda*, and our results will facilitate the screening for and breeding of disease-resistant poplar varieties.

## Results

### Cloning and Sequence Analysis of *PtDef*

**FIGURE 1:**
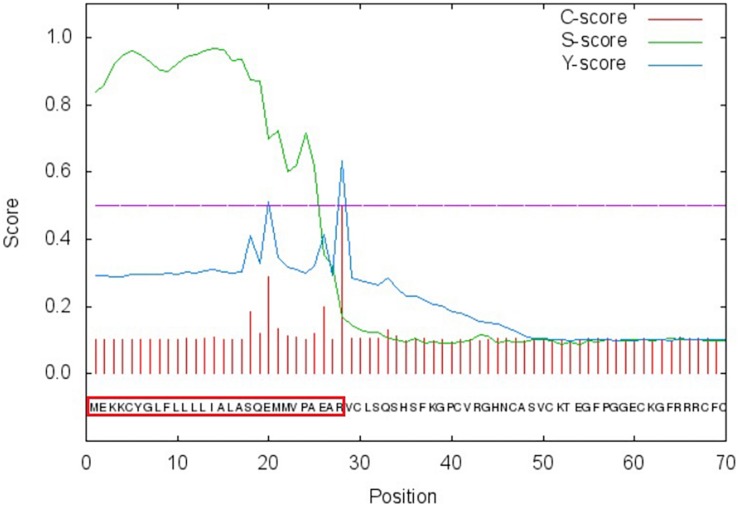
Signal peptide analysis of PtDef. Red box, signal peptide.

We cloned *PtDef* (XP_002325735.1) from *P. trichocarpa* (Tuskan et al., 2006). *PtDef* had a 225-bp open reading frame (ORF) that encoded a 74 amino acid polypeptide (Supplementary Figure S1). The predicted molecular weight and isoelectric point of PtDef were 8.13 kDa and 8.98, respectively. A comparison of the amino acid sequence of PtDef with that of other defensins indicated the presence of eight invariant residues of cysteine (Supplementary Figure S2). The degree of conservation was higher at the C-terminus than at the N-terminus (Supplementary Figure S2). The N-terminus of PtDef has a 27 amino acid signal peptide (Figure 1).

The eight conserved Cys residues in PtDef form four intrachain disulfide bonds: Cys1-Cys8, Cys2-Cys5, Cys3-Cys6, and Cys4-Cys7 (Supplementary Figure S3). The Cys1-Cys8 and Cys2-Cys5 bonds are characteristic of plant defensins (Selsted and Ouellette, 2005). A defensin phylogenetic tree constructed using MEGA5 software showed that PtDef was closely related to a defensin of *Ricinus communis* (XP_025012078.1) (Supplementary Figure S4). In *R. communis* (XP_025012078.1), the function of RcDef was annotated as gamma–thionin, indicating that PtDef may have similar activity.

### Tissue-Specificity and Induction of the Expression of *PtDef*

**FIGURE 2:**
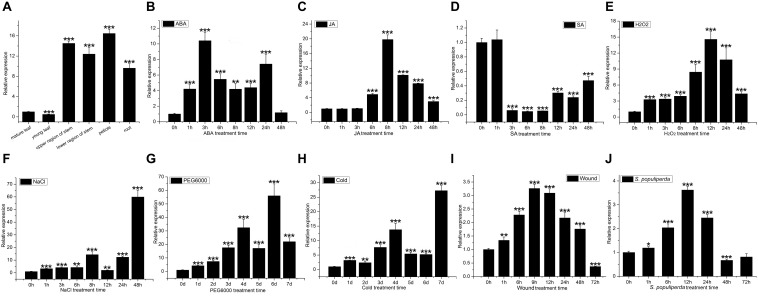
*PtDef* expression in poplar tissues and under abiotic and biotic stresses. **(A)** *PtDef* expression in mature leaves, young leaves, upper region of stems, lower region of stems, petioles, and roots. Student’s *t*-test, ***P < 0.001 compared to the value in mature leaves. Effects on *PtDef* expression of **(B)** abscisic acid (ABA), **(C)** jasmonic acid (JA), **(D)** salicylic acid (SA), **(E)** hydrogen peroxide (H_2_O_2_), **(F)** NaCl, **(G)** PEG_6000_, **(H)** cold, **(I)** wounding, and **(J)** *S*. *populiperda*. Values are means ± standard deviation (SD) of three biological replicates. Student’s *t*-test, ***P < 0.001, **P < 0.01, and *P < 0.05 relative to 0 h.

The expression of *PtDef* was detected by qRT-PCR in all tissues tested, with the highest expression in petioles, followed by stems, roots, and leaves, particularly in young leaves (Figure 2A).

Treatment with 200 μM ABA induced the expression of *PtDef* (Figure 2B); expression peaked at 3 h and subsequently decreased slightly to a level above background. Treatment with 200 μM JA significantly increased *PtDef* expression at 6 h with a peak at 8 h (Figure 2C), followed by a gradual decrease to a level significantly higher than the control upon treatment, as indicated by leakage of cell contents. Treatment with 200 μM SA at 1 h resulted in no statistically significant increase in *PtDef* expression, followed by a decrease to a low level at 3–48 h (Figure 2D). Treatment with 2 mM H_2_O_2_ increased *PtDef* expression at 1 h, followed by a gradual increase to a peak at 12 h and a decrease at 24–48 h to a level significantly higher than the control (Figure 2E). In the presence of 200 mM NaCl, *PtDef* expression peaked at 8 h, decreased, and subsequently increased to a second peak at 48 h (Figure 2F). Under 10% PEG_6000_ treatment, *PtDef* expression increased gradually at days 1–4, decreased slightly at day 5 to a level above that of the control, and subsequently increased to a peak at day 6 (Figure 2G). Under cold treatment, *PtDef* expression peaked at day 4, subsequently decreased slightly to a level above that of the control and reached a higher peak at day 7 (Figure 2H). Under wound stress, *PtDef* expression increased gradually beginning at 1 h to a peak at 9 h, followed by a gradual decrease (Figure 2I). Following inoculation with *S*. *populiperda*, *PtDef* expression increased significantly at 12 h and then decreased (Figure 2J). These results suggest that *PtDef* responds to abiotic and biotic stresses and is involved in a variety of physiological processes.

### Prokaryotic Expression and Purification of Recombinant *PtDef*

**FIGURE 3:**
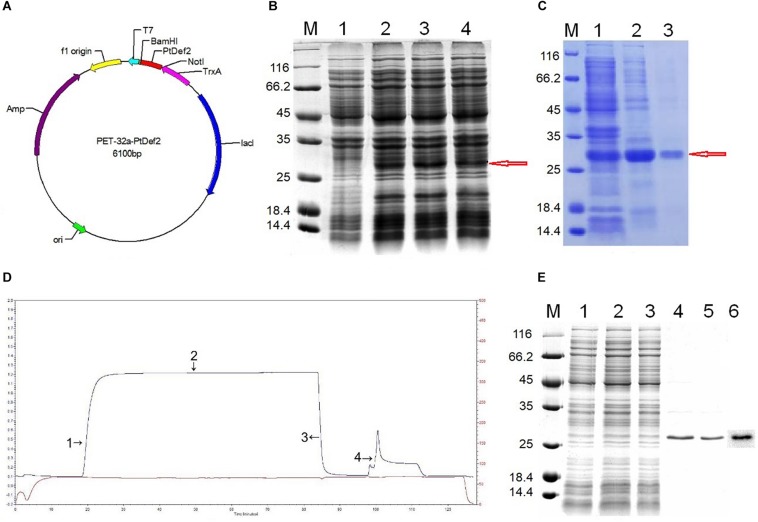
Prokaryotic expression and purification. **(A)** Construction of the expression vector PET-32a-*PtDef*. *PtDef* was inserted into *Bam*HI and *Not*I restriction sites. **(B)** Analysis of the PtDef fusion protein by 12% SDS-PAGE. Lane M, molecular mass marker; Lane 1, negative control (non-induction); Lanes 2–4, colonies 1–3 (induction); red arrow, PtDef fusion protein. **(C)** Analysis of PtDef fusion protein supernatant and sediment by 12% SDS-PAGE. Lane M, molecular mass marker; Lane 1, induction; Lane 2, sediment; Lane 3, supernatant; red arrow, PtDef fusion protein. **(D)** Ni-IDA affinity chromatography of the Trx-PtDef fusion protein using LP Data View. Lanes 1 and 2, flow-through; Lane 3, wash; Lane 4, elution. **(E)** Purification and western blotting of PtDef fusion protein. Lane M, molecular weight marker; Lanes 1 and 2, flow-through; Lane 3, wash; Lanes 4 and 5, elution; Lane 6, western blot of PtDef using a monoclonal antibody against the 6 × His tag.

The ORF of *PtDef* was ligated into PET-32a using the *Not*I and *Bam*HI sites (Figure 3A). Upon SDS-PAGE analysis, IPTG-induced, but not non-induced, *E*. *coli* BL21 (DE3) yielded a band for the Trx-PtDef fusion protein (Figure 3B). SDS-PAGE analysis showed that Trx-PtDef was present in the sediment (Figure 3C, Lane 2) and supernatant (Figure 3C, Lane 3). The presence of supernatant simplified PtDef purification by obviating the need for denaturation and renaturation.

Purification of Trx-PtDef by Ni-IDA resin affinity chromatography (Figure 3D) yielded an outflow peak and an elution peak. Upon SDS-PAGE analysis, a clear band was detected in the effluent and eluent liquid; the protein within this band was identified as Trx-PtDef by western blotting (Figure 3E).

### Antifungal Activity of PtDef

**FIGURE 4:**
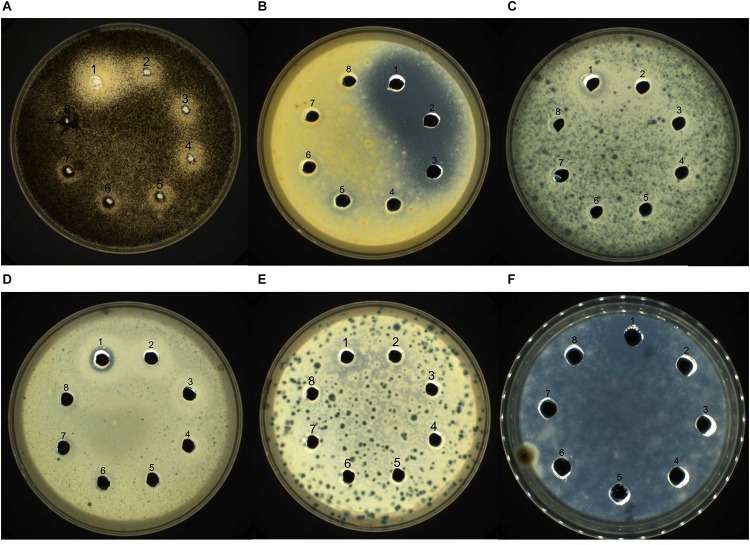
Bioactivity of PtDef against **(A)** *Aspergillus niger*, **(B)** *Alternaria Nees*, **(C)** *Mucor corymbifer*, **(D)** *Marssonina populi*, **(E)** *Rhizopus* sp., and **(F)** *Neurospora crassa*. Lanes 1–7, purified Trx-PtDef fusion protein (40, 20, 10, 5, 2.5, 1.25, and 0.625 μg/ml); Lane 8, 0.9% NaCl (negative control). Each well was loaded with 80 μl of sample.

The antifungal activity of purified recombinant PtDef was assayed *in vitro* (Figure 4) using the agarose diffusion method. Recombinant PtDef inhibited the growth of *A*. *niger*, *A*. *Nees*, *M*. *corymbifer*, *M*. *populi*, *Rhizopus* sp., and *N*. *crassa*, while the growth of the control was normal. The antifungal activity of PtDef exhibited dose dependency and differed among the fungal species tested.

### IPTG-Induced Production of PtDef Inhibits the Growth of *E. coli*

The addition of IPTG for 2–4 h inhibited the growth of *E*. *coli* BL21 (DE3). The OD_600_ value of *E*. *coli* BL21 (DE3) cultures was stable 3–4 h after the addition of IPTG. In contrast, the growth of *E*. *coli* BL21 (DE3) in the absence of IPTG (control) was normal. Therefore, IPTG-induced production of PtDef inhibits growth of *E*. *coli* BL21 (DE3) (Supplementary Figure S5A).

To investigate the mechanism by which PtDef inhibits *E*. *coli* BL21 (DE3), we performed a transcriptome analysis. According to the paradigm of promotion by antibiotics of bacterial autolysis (Supplementary Figure S5B; Kohanski et al., 2007), antibiotics modulate the NAD(H) level by activating the electron transport chain, triggering superoxide production. Superoxide disrupts the iron oxidation balance, leading to the formation of hydroxyl radicals; the destruction of DNA, proteins, and lipids; and ultimately cell death. The expression of 13 of the 26 genes involved in the TCA cycle was altered; seven (*fumC*, *sdhD*, *sdhC*, *gltA*, *sdhB*, *sdhA*, and *sucA*) were upregulated and six (*acnA*, *ydbK*, *frdC*, *frdD*, *frdA*, and *frdB*) were downregulated (Supplementary Figure S6A). Additionally, the expression of 16 of the 41 genes involved in oxidative phosphorylation was altered; six (*cyoA*, *cyoE*, *sdhD*, *sdhC*, *sdhB*, and *sdhA*) were upregulated and 10 (*nuoF*, *nuoE*, *nuoC*, *ppa*, *appC*, *frdC*, *appB*, *frdD*, *frdA*, and *frd B*) were downregulated compared with the control (Supplementary Figure S6B). Moreover, the expression of seven genes related to RNA degradation was altered; four (*rhlB*, *hfq*, *rho*, and *rppH*) were upregulated and three (*groL*, *eno*, and *rne*) were downregulated (Supplementary Figure S7A). The expression of 26 genes (*rplC*, *rplD*, *rplW*, *rplB*, *rpsS*, *rplV*, *rpsC*, *rplP*, *rpsM*, *rpsK*, *rplY*, *rplF*, *rpsD*, *rpsB*, *rpsJ*, *rplX*, *rplM*, *rpsH*, *rpsN*, *rpsE*, *rplR*, *rplK*, *rplA*, *rpsF*, *rpsG*, and *rpsL*) related to the ribosome was downregulated (Supplementary Figure S7B). The expression of two genes related to DNA replication was altered; *dnaE* was downregulated and *rnhA* was upregulated (Supplementary Figure S7C). Some bactericidal antibiotics disrupt bacterial respiration by blocking the entry of pyruvate into the TCA cycle, which slows growth. We found that the expression of 10 genes related to glycolysis/gluconeogenesis (*nuoF*, *nuoE*, *nuoC*, *ppa*, *appC*, *frdC*, *appB*, *frdD*, *frdA*, and *frdB*) was downregulated (Supplementary Figure S8A). Also, *pykA*, the product of which mediates the production of pyruvate in the glycolytic pathway, was downregulated, as was that of the gene encoding pyruvate kinase (*pykF*), indicating suppression of the generation of lactate from pyruvate. Finally, *frdA*, *frdB*, *frdC*, and *frdD* were downregulated, and the transcript levels of genes for succinate acid under fermentation were downregulated (Supplementary Figure S8B). Based on the above, we hypothesized that PtDef induces bacterial autolysis.

### Transcriptome Sequencing Analysis

**FIGURE 5:**
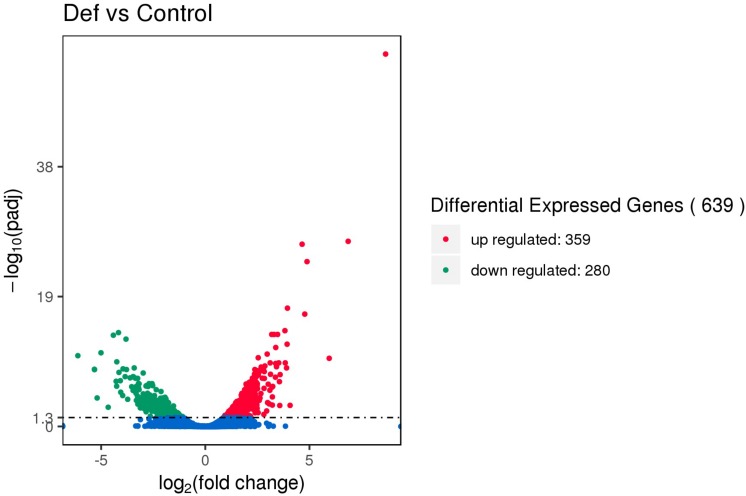
Volcano map of differentially expressed genes (DEGs). The abscissa represents the fold change in expression and the ordinate represents the significance of the change in gene expression. Smaller corrected *P* values indicate larger values of –log(P), i.e., greater statistical significance.

Using Illumina second-generation high-throughput sequencing we generated a total of 24,849,094 raw read pairs. After quality control, 24,340,267 clean read pairs were obtained. Analysis using Bowtie2 and Rockhopper software resulted in the identification of 639 differentially expressed genes (DEGs). Compared with the control, 359 DEGs were upregulated and 280 were downregulated in the experimental group (Figure 5). The 639 unigenes clustered into three expression patterns according to their expression levels in the control and experimental groups (Supplementary Figure S9).

The 639 DEGS were divided into three major GO categories (30 functional groups): biological processes, cellular components, and molecular functions. The three most highly enriched functional groups were cellular metabolic process, biosynthetic process, and cellular biosynthetic process (Supplementary Figure S9). The top 20 enriched pathways (Supplementary Figure S10) included biosynthesis of secondary metabolites, biosynthesis of antibiotics, and microbial metabolism in diverse environments.

The expression of 10 genes related to TCS (narZ, narY, pstS, hyaC, appC, frdC, appB, frdD, frdA, and frdB) was downregulated and that of 12 genes (gltI, arcA, evgA, narP, crp, ompF, NA, fepA, glnL, glnA, baeR, and zraP) was upregulated (Supplementary Table S2). Bacteria survive in hostile environments by regulating their gene pool. TCS is involved in bacterial adaptation to changes in the environment, as well as antibiotic resistance and virulence (Stock et al., 2000; Stephenson and Hoch, 2002). The SOS reaction maintains bacterial growth after damage and is important for sensing and transmitting antibiotic resistance and virulence factors (Beceiro et al., 2013). Based on the transcriptome data, we conclude that PtDef induced by IPTG inhibited *E. coli*, but that *E. coli* adopted strategies such as TCS to regulate bacteria to adapt to the adverse environment. The expression of genes related to the SOS response, such as lexA and RecA (Baharoglu et al., 2010), was not altered. During bacterial drug resistance, the SOS response regulated by RecA is an important pathway for drug resistance (Baharoglu et al., 2010). Together, these findings indicate that TCS is triggered, but that the SOS response fails to trigger, when *E. coli* is maintained in an adverse environment where PtDef inhibits its growth.

### Identification of Transgenic Nanlin895 Lines

We screened 10 transgenic poplar lines throughout the differentiation, bud elongation, and rooting stages. PCR using PtDef-F and PtDef-R specifically amplified 225-bp bands in the transgenic lines, whereas WT poplars had a much larger band (Supplementary Figure S11A). Using 35S and PtDef-R as forward and reverse primers, PCR yielded specific bands from transgenic but not WT poplars (Supplementary Figure S11B), indicating genome integration of *PtDef*. qRT-PCR confirmed that expression of *PtDef* was 2- to 4-fold higher in transgenic poplar than in WT poplar (Supplementary Figure S11C).

### Disease Tolerance of Transgenic Nanlin895 Poplar

**FIGURE 6:**
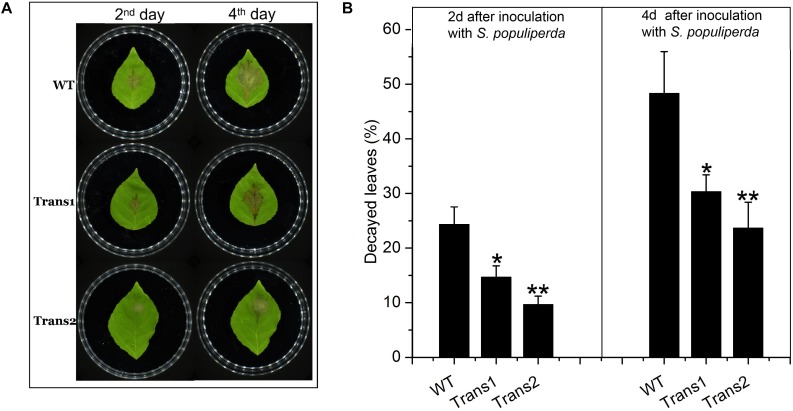
Overexpression of *PtDef* enhances resistance to *S*. *populiperda*. **(A)** Transgenic poplar overexpressing *PtDef* showed fewer lesions than WT poplar following inoculation with *S*. *populiperda*. **(B)** Diseased area of transgenic and WT poplar following inoculation with *S*. *populiperda.* Values are means ± SD of three biological replicates. Student’s *t*-test, **P < 0.01 relative to 0 h.

We evaluated the resistance to *S*. *populiperda* infection of the transgenic (Trans1 and Trans2) and WT poplars. Plaques grew more rapidly on WT poplar leaves than on transgenic poplar leaves at 2–4 days after infection (Figure 6A). On days 2 and 4, spots covered 24.33 ± 3.21% and 14.67 ± 2.08% (*Trans* 1) or 9.67 ± 1.53% (*Trans* 2), and 48.33 ± 7.64% and 30.33 ± 3.06% (*Trans* 1) or 23.67 ± 4.73% (*Trans* 1), of the surface of WT and transgenic poplar leaves, respectively (Figure 6B).

**FIGURE 7:**
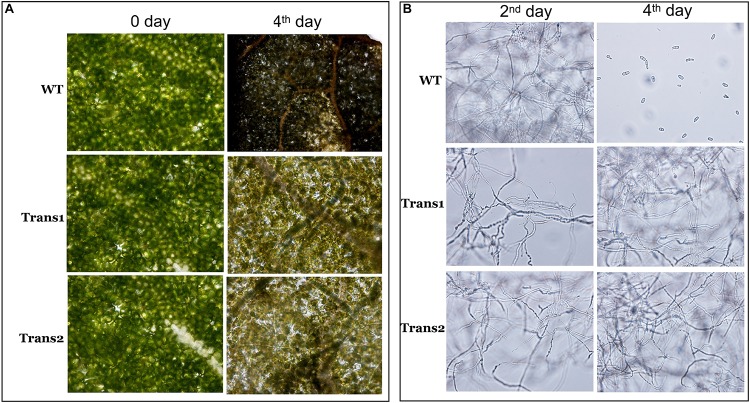
Effects of inoculation with *S*. *populiperda* on disease leaf symptoms. **(A)** Microscopic observation of changes in transgenic and WT leaf internal structure under inoculation with *S*. *populiperda*. **(B)** Microscopic observation of *S. populiperda* generation time under inoculation with *S*. *populiperda*. Three independent experiments were performed.

Evaluation of the damage to poplar leaves caused by *S*. *populiperda* revealed that little damage had been inflicted on the cells of transgenic poplar on day 4, while the cells of WT poplar leaves showed considerable damage at day 4 by microscopy (Figure 7A). The generation time of *S*. *populiperda* was shorter on the leaves of the WT poplar than the transgenic poplar (Figure 7B). Therefore, overexpression of *PtDef* in Nanlin895 poplar enhanced resistance to *S*. *populiperda*.

### Effect of *S. populiperda* on the Expression of Genes Related to SA and JA Pathways

**FIGURE 8:**
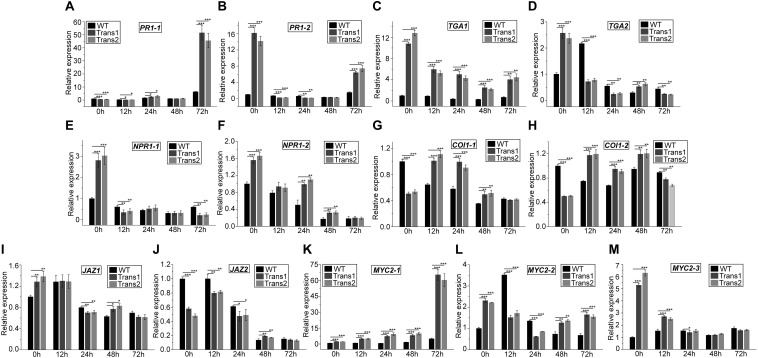
Overexpression of *PtDef* modulates the expression of JA and SA pathway-related genes at 72 h after inoculation of *S*. *populiperda*. The expression pattern at 72 h of **(A)** *PR1-1* (Potri.009G082900), **(B)** *PR1-2* (Potri. T131500), **(C)** *TGA1* (Potri.010G226500), **(D)** *TGA2* (Potri.016G134000), **(E)** *NPR1-1* (Potri.016G040500), **(F)** *NPR1-2* (Potri.006G043400), **(G)** *COI1-1* (Potri.010G192900), **(H)** *COI1-2* (Potri.008G064400), **(I)** *JAZ1* (Potri.010G108200), **(J)** *JAZ2* (Potri.005G214300), **(K)** *MYC2-1* (Potri.006G148800), **(L)** *MYC2-2* (Potri.003G128000), and **(M)** *MYC2-3* (GenBank accession no. XP_024453007.1). Values are means ± SD of three biological replicates. Student’s *t*-test, ***P < 0.001, **P < 0.01, and *P < 0.05 compared to WT poplar.

The expression of *PR1-1* was slightly lower in transgenic poplar than in WT at 0–12 h; at 72 h, the opposite trend was observed, such that *PR1-1* expression was 51.5-fold upregulated in transgenic plants and 6.4-fold upregulated in WT (Figure 8A). *PR1-2* expression was significantly lower at 12–24 h in transgenic poplar than in WT. However, *PR1-2* expression showed the opposite trend at 72 h (Figure 8B). *TGA1* expression was higher in the transgenic poplar than in WT at 0–72 h (Figure 8C). In transgenic poplar, *TGA2* expression was higher at 0 h and 48 h (Figure 8D). *NPR1-1* expression was higher in transgenic poplar than in WT under pre-inoculation, and lower than in WT poplar at 72 h (Figure 8E). *NPR1-2* expression was higher in the transgenic line than in WT at 0–48 h (Figure 8F).

*COI1-1* expression was lower in transgenic poplar than in WT before inoculation with *S*. *populiperda*. Post-inoculation, *COI1-1* expression was higher in transgenic poplar than in WT at 12–48 h (Figure 8G). *COI1-2* expression was higher in the transgenic poplar than in WT at 12–48 h, except at 0 and 72 h (Figure 8H). Post-inoculation, *JAZ1* expression was lower in transgenic poplar than in WT at 24 h (Figure 8I). The expression of *JAZ2* in transgenic poplar was markedly lower than that in WT pre-inoculation. *JAZ2* expression in WT was lower following inoculation with *S*. *populiperda*, whereas in transgenic poplar *JAZ2* expression was higher at 12 h (Figure 8J). The expression of *MYC2-1* was higher in transgenic poplar than in WT at 0–72 h (Figure 8K). *MYC2-2* expression was higher in WT than in transgenic poplar at 12–24 h (Figure 8L). *MYC2-3* expression was highest in WT poplar at 0–12 h (Figure 8M).

Inoculation with *S*. *populiperda* modulated the expression of 13 genes related to SA and JA signal transduction in the transgenic and WT poplars. The expression of *PR1-1* (12–72 h), *NPR1-2*, *TGA1*, and *MYC2-1* in transgenic poplar was higher than that in WT poplar. The expression of *TGA1*, *TGA2*, *NPR1-1*, and *NPR1-2* was downregulated post-inoculation with *S*. *populiperda* in both transgenic and WT poplars, and the magnitude of downregulation was greater in the former.

### Effect of *S. populiperda* on the Expression of ROS-Related Genes and H_2_O_2_ Content

**FIGURE 9:**
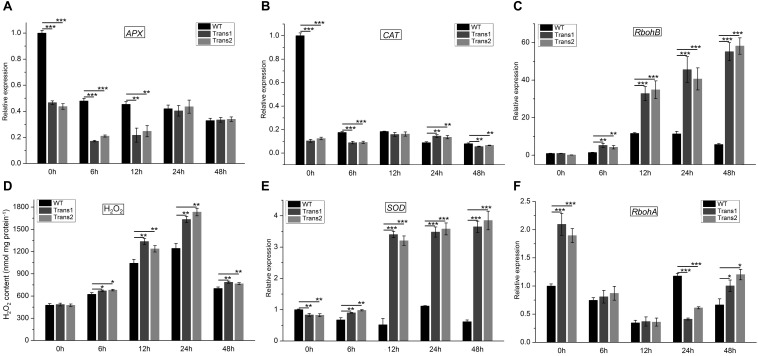
H_2_O_2_ content and expression of genes related to redox metabolism in transgenic and WT poplar leaves pre- and post-inoculation with *S*. *populiperda.* Expression of **(A)** *APX*, **(B)** *CAT*, **(C)** *RbohB* in transgenic and WT poplar post-inoculation with *S*. *populiperda.* **(D)** H_2_O_2_ content of transgenic and WT poplar following inoculation with *S*. *populiperda*. Expression of **(E)** *SOD* and **(F)** *RbohA* in transgenic and WT poplar following inoculation with *S*. *populiperda.* Values are means ± SD of three biological replicates. Student’s *t*-test, ***P < 0.001, **P < 0.01, and *P < 0.05 compared to WT poplar.

H_2_O_2_ exerts a toxic effect on pathogens, participates in lignification, strengthens the cell walls of plants, hinders the penetration of fungi, induces programmed cell death, and acts as a signaling molecule (Heller and Tudzynski, 2011; Lehmann et al., 2015). The H_2_O_2_ content of the transgenic polar was higher than that of WT poplar at 48 h post-inoculation with *S*. *populiperda*. The H_2_O_2_ content of transgenic and WT poplars increased from 0 to 24 h post-inoculation, and then decreased significantly (Figure 9F). Therefore, the greater resistance to *S*. *populiperda* of transgenic than WT poplar may be related to their higher H_2_O_2_ content.

qRT-PCR showed that infection with *S*. *populiperda* downregulated *APX* and *CAT* expression in transgenic and WT poplar at most time points, and *APX* and *CAT* expression was lower in transgenic than in WT poplar (Figures 9A,B). Additionally, *RbohB* expression in transgenic and WT poplar was upregulated at most time points and was markedly higher in transgenic poplar than in WT poplar (Figure 9C). Therefore, compared with WT poplar, transgenic poplar had lower H_2_O_2_ scavenging activity and higher production of H_2_O_2_ (Figure 9D). A high H_2_O_2_ level at the early stage (0–48 h) of infection may induce the expression of defense-related genes or function as a virulence factor (Apel and Hirt, 2004).

Although H_2_O_2_ is important for plant defense responses, excess H_2_O_2_ may contribute to fungal infection. *SOD* expression in transgenic poplar was markedly higher than that in WT poplar post-inoculation with *S*. *populiperda* (Figure 9E). Additionally, *RbohA* expression in transgenic poplar was higher than that in WT poplar except at 24 h (Figure 9F). The high *SOD* expression in transgenic poplar likely serves to prevent excessive accumulation of H_2_O_2_ in leaves. Therefore, the H_2_O_2_ content in transgenic poplar is maintained at a high level, which promotes resistance to *S*. *populiperda* but not to fungal infection.

## Discussion

Growing plants are vulnerable to pathogens and prone to a variety of diseases, which threaten crop yields, the environment, and sustainable agricultural development. Defensins are cationic antimicrobial peptides (AMPs) found in animals and plants and have broad-spectrum bactericidal activity (Thery and Arendt, 2018; Velivelli et al., 2018). In this study, we cloned a defensin gene from *P*. *trichocarpa*. Bioinformatics analysis revealed that *PtDef* had an ORF of 225 bp and encoded a 74 amino acid polypeptide. The N-terminus of PtDef had a signal peptide of 27 amino acids and eight cysteine residues. PtDef has a typical defensin structure with four disulfide bonds composed of eight cysteine residues (García-Olmedo et al., 1998). The defensin of *Nigella Sativa* has a strong inhibitory effect on *Fusarium wilt*, *Botrytis cinerea*, *Fusarium graminearum*, and other fungi (Rogozhin et al., 2011). Tf-gd1, a *Trigonella foenum-graecum* defensin, inhibits the growth of *Thanatephorus cucumeris in vitro* (Olli and Kirti, 2006), whereas the defensin of *Brassica napus* has bacteriostatic activity against *Pyricularia oryzae* and *Xanthomonas oryzae* (Motoshige et al., 2003). Agar diffusion assays revealed that PtDef is active against *A*. *niger*, *A*. *Nees*, *M*. *corymbifer*, *M*. *populi*, *Rhizopus* sp., and *N*. *crassa*. The expression of some plant defensin genes is constitutive and that of others is induced (Mirouze et al., 2006). The expression of the same defensin gene may also differ among varieties of the same species. For example, alfalfa defensin genes are expressed in *Medicago sativa* leaves, flowers, and seeds, but not in roots, while in *Medicago truncatula*, they are expressed in seeds but not in other organs (Hanks et al., 2005). In this study, *PtDef* was expressed in petioles, roots, stems, and leaves of poplar leaves, and was highest in petioles, followed by roots, stems, and leaves. qRT-PCR analysis revealed that *PtDef* responded to a variety of stresses. We speculate that PtDef is involved in a large number of physiological processes.

The addition of IPTG to cultures reduced the growth rate of *E*. *coli* BL21 (DE3). Transcriptome analysis of recombinant *E. coli* BL21 (DE3) before (control) and after induction by IPTG revealed alterations in the expression levels of genes related to the TCA cycle, oxidative phosphorylation, RNA degradation, ribosome, DNA replication, glycolysis/gluconeogenesis, and pyruvate metabolism. Based on antibiotic-induced bacterial autolysis, we speculate that PtDef induces autolysis of *E*. *coli*. Bacterial resistance to traditional antibiotics will render treatment of infection increasingly problematic (Hamilton et al., 2009). Therefore, new antibiotics are urgently needed. AMPs have broad-spectrum antimicrobial activity, immune-regulatory functions, and are important in maintaining homeostasis (Bastos et al., 2018). Therefore, AMPs may be feasible as alternatives to antibiotics. However, some bacteria are resistant to the killing effect of AMPs (Frick et al., 2003), whether they are from the host or administered for therapeutic purposes. Additionally, we found that PtDef modulates the expression of TCS-related genes in *E*. *coli*. Because this system often participates in bacterial adaptation to adverse environments, we preliminarily speculate that *E*. *coli* activates TCS to promote such adaptation.

An improved understanding of the biological characteristics and functions of defensins has enabled progress in the application of defensin-encoding genes. Plant varieties with enhanced disease resistance can be produced by transforming exogenous disease-resistance genes into the plant genome. Transfer of *BjD* from mustard into tobacco and peanut resulted in transgenic tobacco resistant to *Phytophthora infestans* and *Fusarium moniliforme* and transgenic peanut resistant to late peanut leaf spot (Anuradha et al., 2008). Rice transformed with the *DmAMPI* gene of *Dahlia merckii* were resistant to infection by *Magnaporthe oryzae* and *Rhizoctonia solani* (Jha et al., 2009). In this study, we used the *Agrobacterium* infection method to transfer *PtDef* into Nanlin895 poplar (*Populus* × *euramericana* cv. Nanlin895). We assessed the rate of lesion development on leaves, degree of damage to leaves, and the generation time of *S*. *populiperda*. We found that transgenic poplar exhibited strong resistance to *S*. *populiperda*.

The plant disease resistance and defense response signaling pathways are termed the SA, ET, and JA pathways; these pathways are interconnected (Koornneef et al., 2008; Islam et al., 2019). They differ in the types of exogenous signals, special signal transduction regulators, types of activated effector genes, and groups of resistant pathogens. However, there are some cross-links among these signaling pathways (Mur et al., 2006; Yasuda et al., 2008). Post-inoculation with *S*. *populiperda*, *TGA1* and *NPR1-2* expression in transgenic poplar was significantly higher than that in WT poplar. *PR1-1* expression at 12, 24, and 72 h in transgenic poplar increased significantly and was significantly higher than that in WT poplar. The regulatory protein *NPR1* is a key cross-point in signal transduction networks composed of various disease-resistance–signal-transduction pathways. *NPR1* acts downstream of SA and interacts with TGA-like transcription factors that bind to the SA response elements in the *PR1* promoter, regulating its expression (Kinkema et al., 2000; Chern et al., 2001; Fan and Dong, 2002). The disease-related protein PR1 triggers production of SAR, inducing broad-spectrum disease resistance (Balint-Kurti, 2019). *NPR1-1*, *NPR1-2*, *TGA1*, and *TGA2* expression in transgenic poplar was significantly higher than that in WT poplar before infection, indicating greater SA accumulation pre-inoculation with *S*. *populiperda*. TGA, NPR1, and PR1 are signal molecule related genes that open the SA signal transduction pathway, causing SAR to be more strongly induced in transgenic poplar. This may explain the strong disease resistance of transgenic poplar.

The expression of *MYC2-1*, *MYC2-2*, and *MYC2-3* was high in transgenic poplar pre-inoculation with *S*. *populiperda*. *MYC2* and other transcription factors are produced in large quantities in plants at certain developmental stages or under conditions of stress. *MYC2* upregulates the expression of the injury- and JA-activated genes *VSP* and *LOX* in a COI1-dependent manner, initiating the transcription of resistance-related genes (Cheong et al., 2002). Therefore, the high expression of *MYC2-1*, *MYC2-2*, and *MYC2-3* in transgenic poplar at the early stage of stress may trigger the transcription of JA-related genes, thus enhancing disease resistance. The SA and JA signaling pathways exert both antagonistic and synergistic effects. JA inhibits the expression of *PR1*, a marker gene involved in the SA signaling pathway (Pieterse et al., 2009). However, Zehnder et al. (2001) observed that JA-mediated ISR induced by *Erwinia tracheiphila* did not affect the expression of *PR1* in the field. In the present study, expression of *PR1-1* in transgenic poplar was lower than that in WT poplar, and *PR1-2* expression in transgenic poplar was higher than that in WT poplar before *S*. *populiperda* infection. However, *PR1-1* and *PR1-2* expression in transgenic poplar at 12 h was lower than that in WT poplar, and *vice versa* at 72 h. Therefore, *PR1-1* expression was downregulated before infection but was upregulated at 24 and 72 h. *PR1-2* expression also increased at 72 h. Coronatine-insensitive 1 (COI1) is a regulatory signal switch in the JA signaling pathway and is involved in JA metabolism (Feys and Parker, 2000; Shen et al., 2018). In the present study, *COI1-1* and *COI1-2* expression in transgenic poplar was lower than that in WT poplar before infection. *COI1-1* and *COI1-*2 expression in the transgenic poplar was higher than that in the WT poplar at 12–48 h. This may be due to the accumulation of more SA signaling molecules in transgenic poplar, which inhibited *COI1* expression. The strong resistance of transgenic poplars to *S*. *populiperda* may be due to the upregulation of *PR1-1* and *MYC2-1* and downregulation of *JAZ1*, *COI1-1*, and *COI1-2*, leading to activation of the SA and JA signaling pathways. The signaling pathways that control poplar disease resistance warrant further investigation.

The rapid generation of ROS is termed an oxidative burst and is an early response of plants to pathogens. ROS include superoxide, hydrogen peroxide, and singlet oxygen, and are produced by plants under stresses such as cold, drought, salt, and pathogen invasion. Rapid generation of ROS is an important defense response of plants to pathogens (Torres et al., 2006). ROS play an important role in plant defense: upon pathogen invasion of resistant plants, the production of oxygen-decomposing enzymes such as APX and CAT is inhibited (Klessig et al., 2000), increasing ROS production and inducing an HR reaction (Melillo et al., 2011). In the present study, *APX* and *CAT* expression in transgenic poplar was significantly lower than that in WT poplar at most time points. Additionally, the H_2_O_2_ content of transgenic poplar was significantly higher than that of WT poplar, and the generation time of *S*. *populiperda* on the leaves of transgenic poplar was significantly longer than that on the leaves of WT poplar, suggesting that the transgenic poplar responded to *S*. *populiperda* by increasing H_2_O_2_ production. The production of ROS in plants induces allergic reactions, in some cases leading to cell death (Fonseca and Mysore, 2019). In plants, excess H_2_O_2_ can cause cell death (Siddique et al., 2014). SOD, CAT, and GST are key enzymes in plant oxygen metabolism, which eliminates excess ROS produced in response to pathogens (Foyer and Shigeoka, 2011). In the present study, *SOD* expression was higher in transgenic than in WT poplar, suggesting higher levels of antioxidants. Based on these findings, we speculate that the ROS content of transgenic poplars must be maintained at a certain level to resist pathogen invasion and prevent damage to the plant.

In conclusion, we cloned a defensin gene from *P*. *trichocarpa* and named it *PtDef* (GenBank accession number XP_002325735.1). Its ORF was 225 bp and encoded a 74 amino acid protein. Phylogenetic analysis revealed that *PtDef* is closely related to *RcDef* of *R*. *communis* (XP_025012078.1). Also, *PtDef* was expressed in all tissues of poplar, and its expression was induced by exogenous biotic and abiotic stresses. Additionally, PtDef inhibited the growth of several fungi. To investigate the mechanism by which PtDef inhibits *E. coli* BL21 (DE3), we performed transcriptome analysis; the results indicated that PtDef induced *E*. *coli* BL21 (DE3) autolysis. Overexpression of *PtDef* in Nanlin895 poplar enhanced its resistance to *S*. *populiperda*. We also explored the reasons for the pathogen resistance of transgenic poplar. The expression of genes related to JA and SA differed between transgenic and WT poplar infected with *S*. *populiperda*. Differences in the expression of genes related to SA and JA signaling may modulate the activity of those pathways. Further research is needed to investigate the control of the signaling pathways involved in the disease resistance of poplar.

## Materials and Methods

### Plant Culture and Treatments

We cultured *P. trichocarpa* and Nanlin 895 under long-day conditions (16-h light/8-h dark) in 1/2 Murashige and Skoog (MS) medium (pH 5.8) at 23°C and 74% humidity. We extracted RNA of young and mature leaves, upper and lower stems, petioles, and roots using a Plant RNA Extraction Kit (Biomiga, Inc.). Leaves of WT poplar seedlings grown for 3 months were treated with 200 mM NaCl, 200 μM ABA, 200 μM SA, 200 μM JA, or 2 mM H_2_O_2_ and sampled at 0, 1, 3, 6, 8, 12, 24, and 48 h. WT poplar seedlings were treated with Macrogol 6000 (PEG_6000_) and at 4°C (cold stress), and sampled at 0, 1, 2, 3, 4, 5, 6, and 7 days. We wounded poplar leaves at 23°C and samples were taken at 0, 6, 12, 24, 48, and 72 h. Leaves of poplar seedlings were inoculated with *S*. *populiperda* on potato dextrose agar (PDA) (Zhu et al., 2019) and sampled at 0, 6, 12, 24, 48, and 72 h; a blank PDA plate was used as control. RNA was then extracted from the leaves using a Plant RNA Extraction Kit (Biomiga, Inc.).

### Gene Cloning and Sequence Analysis

First-strand cDNA was synthesized using a Reverse Transcription Kit (TaKaRa, Japan) in reactions comprising 200 ng RNA template, 1 μl enzyme mix 1, 1 μl random 6-mers, 1 μl OligodT primer, 4 μl 5 × PrimeScript buffer, and RNase-free ddH_2_O to a volume of 20 μl. PCR was performed at 42°C for 30 min and 85°C for 5 s, yielding 20 μl cDNA.

The National Center for Biotechnology Information (NCBI) database^1^ was used to search for defensin-encoding genes in the *P*. *trichocarpa* genome and to design primers (Supplementary Table S1). Each reaction comprised 2 μl cDNA (template), 2 μl of the forward and reverse primers (10 μM), 0.5 μl raq, 4 μl dNTPs (2.5 mM), 5 μl 10 × PCR buffer (Mg^2+^), and sterile water to a volume of 50 μl. PCR was performed as follows: 95°C for 10 min, followed by 35 cycles of 95°C for 0.5 min, 58°C for 0.5 min, 72°C for 0.5 min, and 72°C for 10 min. PCR products were identified by electrophoresis in 1% agarose gel and purified using a commercial kit (Axygen, Sunzhou, China). The PCR product was ligated into the PEASY-T3 vector (TransGen Biotech, Beijing, China) and transformed into competent cells of *E*. *coli* TransT1 (TransGen Biotech). Positive clones were screened for sequencing by Nanjing Genscript Co. (Nanjing, China).

Protparam^2^ was used to analyze the physicochemical properties of the amino acids of PtDef, and the Signal server^3^ was used for signal peptide analysis. A homology search for defensins was performed in the NCBI database, and a phylogenetic tree was constructed using the MEGA 5.0 software.

### Analysis of *PtDef* Expression

We designed qRT-PCR primers specific for *PtDef* (Supplementary Table S1) using the Primer 3.0 software. *PtActin* (GenBank accession no. XM006370951.1) (Supplementary Table S1) was used as an internal reference, and qRT-PCR was performed using a StepOnePlus real-time fluorescence qPCR instrument (Applied Biosystems, Foster City, CA, United States). The qRT-PCR reactions comprised 10 μl SYBR Green, 1 μl forward primer (10 μmol), 1 μl reverse primer (10 μmol), 1 μl cDNA (template), and sterile water to a volume of 20 μl. qRT-PCR involved 40 cycles of pre-denaturation at 95°C for 10 min, denaturation at 95°C for 10 s, and annealing at 60°C for 30 s.

### Construction of PET-32a-*PtDef* and Recombinant Plasmid Expression

Primers (Supplementary Table S1) were designed and synthesized based on the *Bam*HI and *Not*I restriction sites in the PET-32a plasmid. The restriction enzymes *Bam*HI and *Not*I (TaKaRa) were used to digest the PCR product and PET-32a plasmid, respectively, followed by ligation using T4 ligase (TaKaRa). Positive clones were electrophoresed in 1% agarose gel and sequenced.

The plasmid pET32a-*PtDef* was transformed into *E*. *coli* BL21 (DE3) (Solarbio, China). A single colony was selected on a plate containing 50 μg/ml ampicillin and cultured in liquid medium containing 50 μg/ml ampicillin. The expression of pET32a-*PtDef* in *E*. *coli* BL21 (DE3) was induced by 1 mmol/L isopropyl-β-D-1-thiogalactopyranoside (IPTG) in 100 ml Luria-Bertani (LB) medium for 4 h at 30°C. Cells were harvested by centrifugation at 5000 rpm for 15 min, resuspended in 10 ml binding buffer (20 mM Tris-base, 500 mM NaCl, and 20 mM imidazole), frozen at −20°C, and thawed in an ice bath; lysozyme was then added for a final volume of 1 mg/ml Next, the mixture was placed on ice for 30 min, Triton X-100 was added, and the concentration of the solution was diluted to 0.1%. DNase and RNase (5 μg/ml) were added and the mixture was incubated at 4°C for 10 min. Finally, DL-dithiothreitol (DTT) was added to a concentration of 1 mmol/L, the mixture was centrifuged, and the supernatant was decanted and stored at 4°C.

His-PtDef was purified by His-label affinity chromatography at a flow rate of 0.5 ml/min and a pressure of 0.25 MPa; the UV280 signal was set to zero. Samples were added after the baseline had stabilized. Next, wash buffer (20 mM Tris-base, 500 mM NaCl, and 50 mM imidazole) was added until the baseline returned to zero and elution buffer (20 mM Tris-base, 500 mM NaCl, and 250 mM imidazole) was applied to elute the target protein. The fractions recovered were evaluated by 12% SDS-PAGE.

#### Antifungal Activity Assay

*Aspergillus niger*, *Alternaria Nees*, *Mucor corymbifer*, *Marssonina populi*, *Rhizopus* sp., and *Neurospora crassa* were inoculated on PDA and activated in a 25°C incubator. The fungi were resuspended in 0.9% NaCl (pH 7.0) and transferred to liquid PDA, which was poured into Petri dishes. After setting, holes were made in the PDA. We dissolved 120 μl PtDef in 0.9% NaCl (pH 7.0) to 40, 20, 10, 5, 2.5, 1.25, and 0.625 μg/ml and added the solutions to PDA in triplicate. PDA containing 0.9% NaCl (pH 7.0) was used as a negative control. The plates were incubated at 25°C for 72 h until observation of mycelia.

### Growth of *E. coli* BL21 (DE3)

Recombinant *E*. *coli* BL21 (DE3) was activated on LB agar containing 50 μg/ml ampicillin. Monoclones were cultured in LB containing 50 μg/ml ampicillin overnight at 37°C with shaking at 220 rpm. The following day, recombinant *E*. *coli* BL21 (DE3) was transferred to fresh LB at a 1:100 dilution and cultured to an OD_600_ of 0.4, after which IPTG was added to 1 mM. OD_600_ was then evaluated at 30-min intervals. A culture not induced by IPTG was used as the control.

### Transcriptome Analysis of *E. coli* BL21 (DE3)

We performed transcriptome analysis of recombinant *E. coli* BL21 (DE3) before (control) and after PtDef induction by IPTG. RNA was extracted from *E*. *coli* BL21 (DE3) using an RNA Extraction Kit (Biomiga, Inc.) its integrity was investigated by electrophoresis in 2% agarose gel. RNA concentration and purity were determined using Nanodrop and Qubit instruments, respectively. The *E*. *coli* transcriptome was sequenced by Allwegene Bioinformatics Technology Co., Ltd. (Beijing, China). Data filtering and quality evaluation were performed, and the transcripts of clean reads were spliced. We used the Nr, Pfam, eggNOG, Swiss-prot, Kyoto Encyclopedia of Genes and Genomes (KEGG), and Gene Ontology (GO) databases for subsequent analyses.

### Construction and Transformation of an Overexpression Plasmid

Forward and reverse primers (Supplementary Table S1) were designed according to the gateway entry vector pENTR/D-TOPO (Invitrogen, Carlsbad, CA, United States). Following amplification by PCR, the BP reaction was performed to construct the plasmid pENTR-PtDef by homologous recombination. Next, the recombinant plasmid pGWB9-*PtDef* (pGWB9 accession no. AB289772) was produced by LR reaction based on LR Clonase II and transformed into *A*. *tumefaciens* EHA105 (Solarbio, China).

Nanlin895 poplar (*Populus* × *euramericana* cv. Nanlin895) transformation was performed as follows. Nanlin895 poplar leaves and petioles were precultured for 3 days on differentiation MS medium (pH 5.8). *A*. *tumefaciens* EHA105 harboring pGWB9-PtDef was incubated at 28°C to an OD of 0.8, harvested by centrifugation at 5000 rpm for 15 min, resuspended in liquid sucrose-free MS medium, and used to infect Nanlin895 poplar leaves and petioles. Finally, Nanlin895 poplar leaves and petioles were transferred to differentiation MS medium and resistant plants were obtained by screening on bud elongation MS medium and screening rooting MS medium (1/2 MS medium) (Movahedi et al., 2015, 2018).

Total DNA of WT and transgenic poplars was extracted using a DNA Extraction Kit (Biomiga, Inc.) and amplified by PCR using PtDef-F and PtDef-R, and 35S (Supplementary Table S1) and PtDef-R, as forward and reverse primers, respectively. Total RNA of WT and transgenic poplars was extracted using an RNA Extraction Kit (Biomiga, Inc.) transcribed into cDNA, and subjected to qRT-PCR as described above.

### Analysis of *Septotinia populiperda* Resistance

*Septotinia populiperda* was cultured on PDA at 25°C for 7 days. Leaves were moisturized using moist sterile degreased cotton. Holes were made in leaves of 4-month-old WT and transgenic poplars using a hole punch and inoculated with *S. populiperda*. Leaves were cultured for 24 h in the dark at 95% humidity and 23°C, and transferred to normal conditions (16 h light/8 h dark, 23°C, 74% humidity). Infection was monitored daily; the expanded range of *S. populiperda* in leaves was measured, and the degree of leaf damage *S. populiperda* growth was observed by microscopy. Analysis of genes related to the JA and SA signaling pathways; APX, CAT, SOD, RbohA, and RbohB expression levels; and H_2_O_2_ content.

*PR1*, *NPR1*, and *TGA* (related to SA signal transduction) and *JAZ*, *COI1*, and *MYC2* (related to JA signal transduction) were amplified by qRT-PCR using the primers listed in Supplementary Table S1. The effects of *S*. *populiperda* infection on the expression of 13 genes were analyzed by qRT-PCR. Expression of the genes encoding ROS-scavenging enzymes *APX*, *CAT*, and *GST* and ROS-producing enzymes *RbohA* and *RbohB* were analyzed by qRT-PCR as described above using the primers listed in Supplementary Table S1.

H_2_O_2_ content was determined as previously described (Gay et al., 1999). Leaf samples inoculated or not with *S*. *populiperda* were collected at 0, 6, 12, 24, and 48 h and placed in liquid nitrogen; 1.5 ml of 500 mM boric acid buffer (pH 8.4) was added. Samples were then mixed gently in an ice bath for 10 min and centrifuged at 10,000 rpm for 20 min at 4°C. Sediments were removed and supernatants were collected, and 1 ml of a solution containing 25 mM ferrous sulfate, 100 mM sorbitol, 0.125 mM xylenol orange, 25 mM ammonium sulfate, and 100 mM sulfuric acid was added. A standard curve was generated from H_2_O_2_ standards at 560 nm using a Genesys 10S UV-vis spectrophotometer (Thermo Scientific, Waltham, MA, United States).

## Data Availability Statement

The raw RNA sequencing data were deposited in the NCBI Sequence Read Archive (SRA) with the accession number SRR9126592.

## Author Contributions

HW and AM designed the experiments. HW and AM wrote the manuscript. HW, AM, CX, WS, PW, and DL read and reviewed the manuscript. TY and QZ supervised this project. QZ funded this project.

## Conflict of Interest

The authors declare that the research was conducted in the absence of any commercial or financial relationships that could be construed as a potential conflict of interest.

## Footnotes

1 http://www.ncbi.nlm.nih.gov/gorf/gorf.html

2 http://web.expasy.org/protparam

3 http://www.cbs.dtu.dk/services/SignalP/

## Supplementary Material

FIGURE S1 Nucleotide and deduced amino acid sequences of PtDef (GenBank accession no. XP_002325735.1). The ATG (initiation codon) is boxed and the TAG (termination codon) is indicated by a five-pointed star.

FIGURE S2 Amino acid sequence alignment of several plant defensins using ClustalW; red arrow, cysteine residues.

FIGURE S3 Amino acid composition distribution of PtDef. Connections between cysteine residues represent disulfide bonds.

FIGURE S4 Phylogenetic analysis of *PtDef*. The phylogenetic tree was constructed using the neighbor-joining method with MEGA 5.1 software and bootstrapped 1000 times. The GenBank accession numbers of the *Def* sequences are: *Ricinus communis* (XM_025156310.1), *Juglans regia* (XM_018955615.1), *Morus notabilis* (XM_024165652.1), *Quercus suber* (XM_024039378.1), *Nicotiana tomentosiformis* (XM_009592617.2), *Cephalotus follicularis* (BDDD01004928.1), *Nicotiana sylvestris* (XM_009781749.1), *Nicotiana attenuata* (XM_019404257.1), *Durio zibethinus* (XM_022879395.1), *Theobroma cacao* (XM_018119227.1), *Ziziphus jujuba* (XM_016024166.2), *Manihot esculenta* (XM_021763356.1), *Medicago truncatula* (XM_013606172.2), *Trifolium subterraneum* (DF973199.1), *Olea europaea var*. *Sylvestris* (XM_022990840.1), *Arachis duranensis* (XM_016100210.2), *Cajanus cajan* (XM_020358126.1), *Nicotiana tabacum* (XM_016595723.1), *Chenopodium quinoa* (XM_021877694.1), *Momordica charantia* (XM_022290632.1), *Capsicum annuum* (XM_016700063.1), *Citrus sinensis* (XM_025094762.1), *Artemisia annua* (PKPP01003814.1), and *Arabidopsis thaliana* (NP_201171.1).

FIGURE S5 **(A)** Growth curve of *E*. *coli* BL (DE3) induced or not by IPTG (Student’s *t*-test, ^∗∗∗^*P* < 0.001 and ^∗∗^*P* < 0.01). **(B)** Mechanism by which antibiotics promote autolysis of bacteria.

FIGURE S6 **(A)** Kyoto Encyclopedia of Genes and Genomes (KEGG) pathway annotation of differentially expressed genes (DEGs) related to the TCA cycle; *acnA*, *ydbK*, *frdC*, *frdD*, *frdA*, and *frdB* were downregulated and *fumC*, *sdhD*, *sdhC*, *gltA*, *sdhB*, *sdhA*, and *sucA* were upregulated. **(B)** KEGG pathway annotation of DEGs related to oxidative phosphorylation; *nuoF*, *nuoE*, *nuoC*, *ppa*, *appC*, *frdC*, *appB*, *frdD*, *frdA*, and *frdB* were downregulated and *cyoA*, *cyoE*, *sdhD*, *sdhC*, *sdhB*, and *sdhA* were upregulated.

FIGURE S7 **(A)** KEGG pathway annotation of DEGs related to RNA degradation; *groL*, *eno*, and *rne* were downregulated and *rhlB*, *hfq*, *rho*, and *rppH* were upregulated. **(B)** KEGG pathway annotation of DEGs related to the ribosome; *rplC*, *rplD*, *rplW*, *rplB*, *rpsS*, *rplV*, *rpsC*, *rplP*, *rpsM*, *rpsK*, *rplY*, *rplF*, *rpsD*, *rpsB*, *rpsJ*, *rplX*, *rplM*, *rpsH*, *rpsN*, *rpsE*, *rplR*, *rplK*, *rplA*, *rpsF*, *rpsG*, and *rpsL* were downregulated. **(C)** KEGG pathway annotation of DEGs related to DNA replication; *dnaE* was downregulated and *rnhA* was upregulated.

FIGURE S8 **(A)** KEGG pathway annotation of DEGs related to glycolysis/gluconeogenesis; *nuoF*, *nuoE*, *nuoC*, *ppa*, *appC*, *frdC*, *appB*, *frdD*, *frdA*, and *frdB* were downregulated. **(B)** KEGG pathway annotation of DEGs related to pyruvate metabolism; *poxB*, *NA*, *pykF*, *aldB*, *pykA*, *ydbK*, *frdC*, *frdD*, *frdA*, *frdB*, and *pflB* were downregulated.

FIGURE S9 KEGG pathway annotations of DEGs.

FIGURE S10 Scatter plot of KEGG pathway enrichment. The abscissa is the enrichment factor of the pathway, the ordinate is the name of the pathway, the number of different genes in the pathway is indicated by the size of the point, and the size of the *P*-value is indicated by the color of the point (the smaller *P*-value, the closer the color to red).

FIGURE S11 Molecular identification of regenerated *PtDef* transgenic plants. **(A)** Identification of *PtDef* in the genome of transgenic and WT poplar by PCR using the PtDef-F and PtDef-R forward and reverse primers, respectively. Lane M, molecular mass marker; Lane 1, negative control (genome of WT poplar as the template); Lanes 2–11, transgenic lines 1–10 (Trans1–Trans10). **(B)** Identification of *PtDef* in the genome of transgenic and WT poplar by PCR using the 35S promoter as the forward primer and PtDef-R as the reverse primer. Lane M, molecular mass marker; Lane 1, negative control (WT poplar genome as the template); Lanes 2–11, transgenic lines 1–10 (Trans1–Trans10). **(C)** Analysis of *PtDef* expression in transgenic and WT poplar by real-time RT-PCR. Values are means ± SD of three biological replicates. Student’s *t*-test, ^∗∗∗^*P* < 0.001 compared to WT poplar.

TABLE S1 Primers used in this study.

TABLE S2 Kyoto Encyclopedia of Genes and Genomes (KEGG) pathway annotation of differentially expressed genes (DEGs).
