# Supplementary material for: Characterization, Expression Profiling, and Functional Analysis of PtDef, a Defensin-Encoding Gene From Populus trichocarpa
Source: Front Microbiol. 2020 Feb 7;11:106. doi: 10.3389/fmicb.2020.00106 (PMC7018670; doi:10.3389/fmicb.2020.00106)
Supplement: FIGURE S1 — Nucleotide and deduced amino acid sequences of PtDef (GenBank accession no. XP_002325735.1). The ATG (initiation codon) is boxed and the TAG (termination codon) is indicated by a five-pointed star. [file Data_Sheet_1.pdf]

Supplementary Material

Supplementary Figures

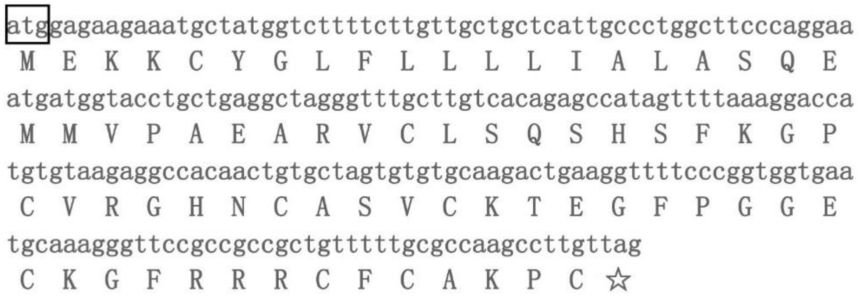

Supplementary Figure 1. Nucleotide and deduced amino acid sequences of PtDef (GenBank accession no. XP_002325735.1). The ATG (initiation codon) is boxed and the TAG (termination codon) is indicated by a five-pointed star.

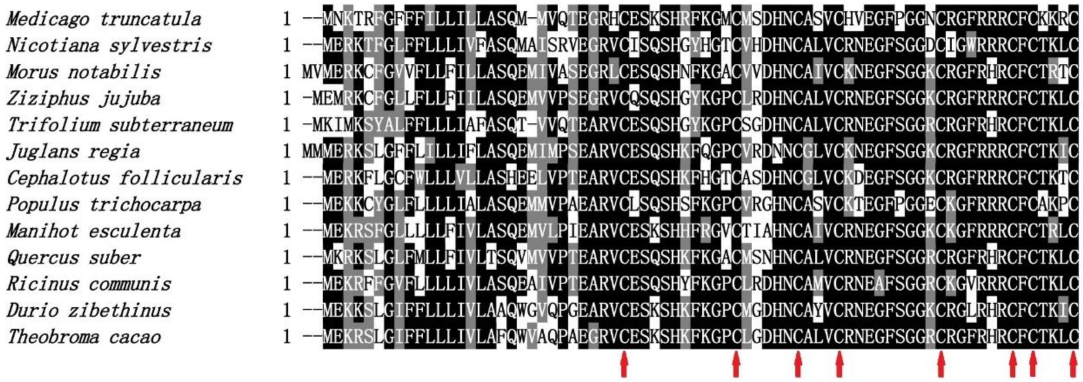

Supplementary Figure 2. Amino acid sequence alignment of several plant defensins using ClustalW; red arrow, cysteine residues.

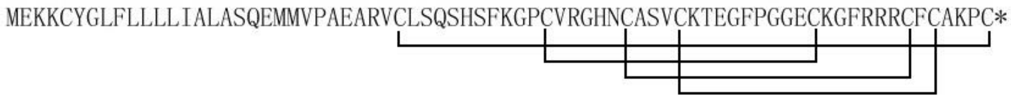

Supplementary Figure 3. Amino acid composition distribution of PtDef. Connections between cysteine residues represent disulfide bonds.

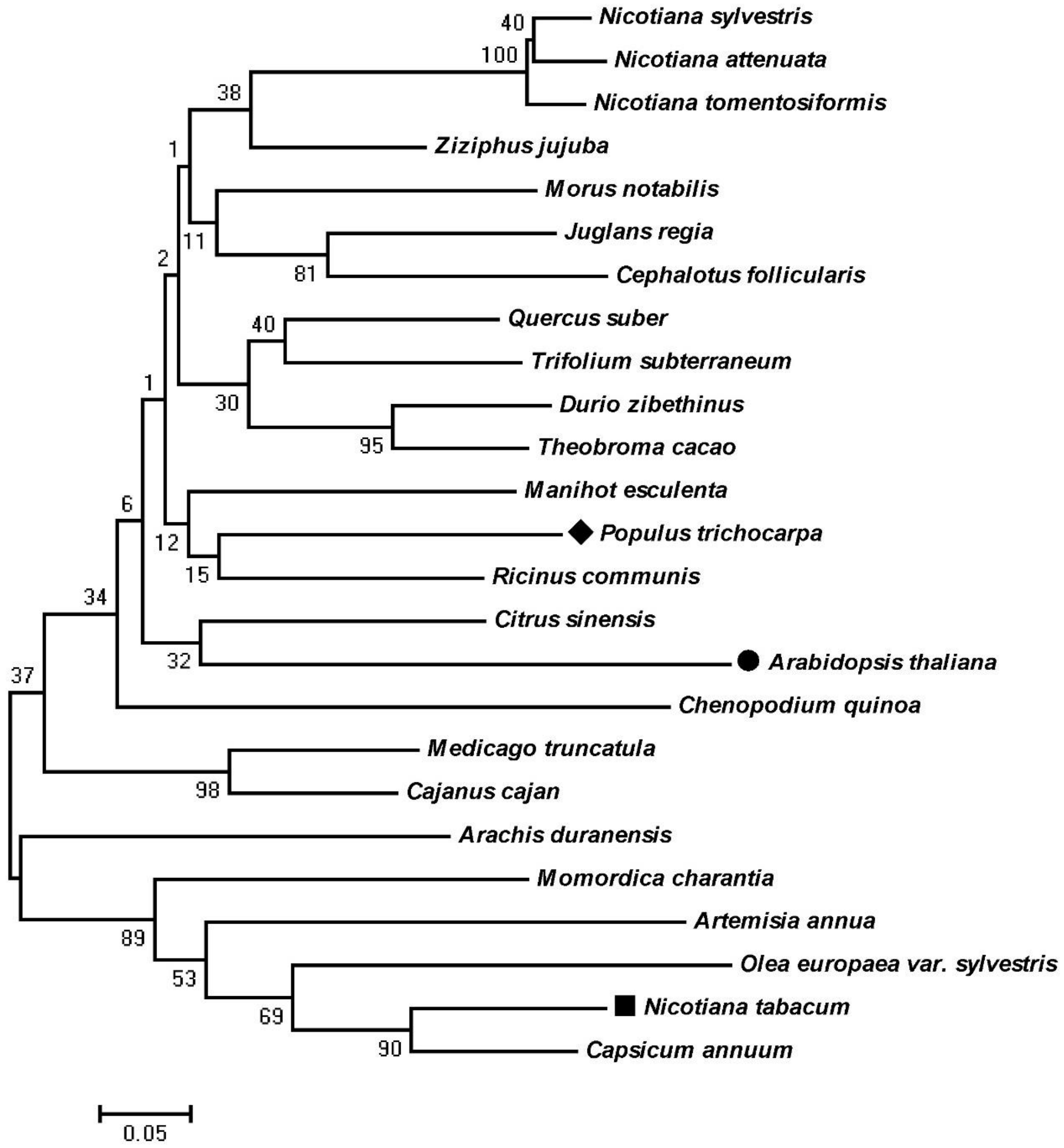

Supplementary Figure 4. Phylogenetic analysis of *PtDef*. The phylogenetic tree was constructed using the neighbor-joining method with MEGA 5.1 software and bootstrapped 1000 times. The GenBank accession numbers of the *Def* sequences are: *Ricinus communis* (XM_025156310.1), *Juglans regia* (XM_018955615.1), *Morus notabilis* (XM_024165652.1), *Quercus suber* (XM_024039378.1), *Nicotiana tomentosiformis* (XM_009592617.2), *Cephalotus follicularis* (BDDD01004928.1), *Nicotiana sylvestris* (XM_009781749.1), *Nicotiana attenuata* (XM_019404257.1), *Durio zibethinus* (XM_022879395.1), *Theobroma cacao* (XM_018119227.1), *Ziziphus jujuba* (XM_016024166.2), *Manihot esculenta* (XM_021763356.1), *Medicago truncatula* (XM_013606172.2), *Trifolium subterraneum* (DF973199.1), *Olea europaea var*. *Sylvestris* (XM_022990840.1), *Arachis duranensis* (XM_016100210.2), *Cajanus cajan* (XM_020358126.1), *Nicotiana tabacum* (XM_016595723.1), *Chenopodium quinoa* (XM_021877694.1), *Momordica charantia* (XM_022290632.1), *Capsicum annuum* (XM_016700063.1), *Citrus sinensis* (XM_025094762.1), *Artemisia annua* (PKPP01003814.1), and *Arabidopsis thaliana* (NP_201171.1).

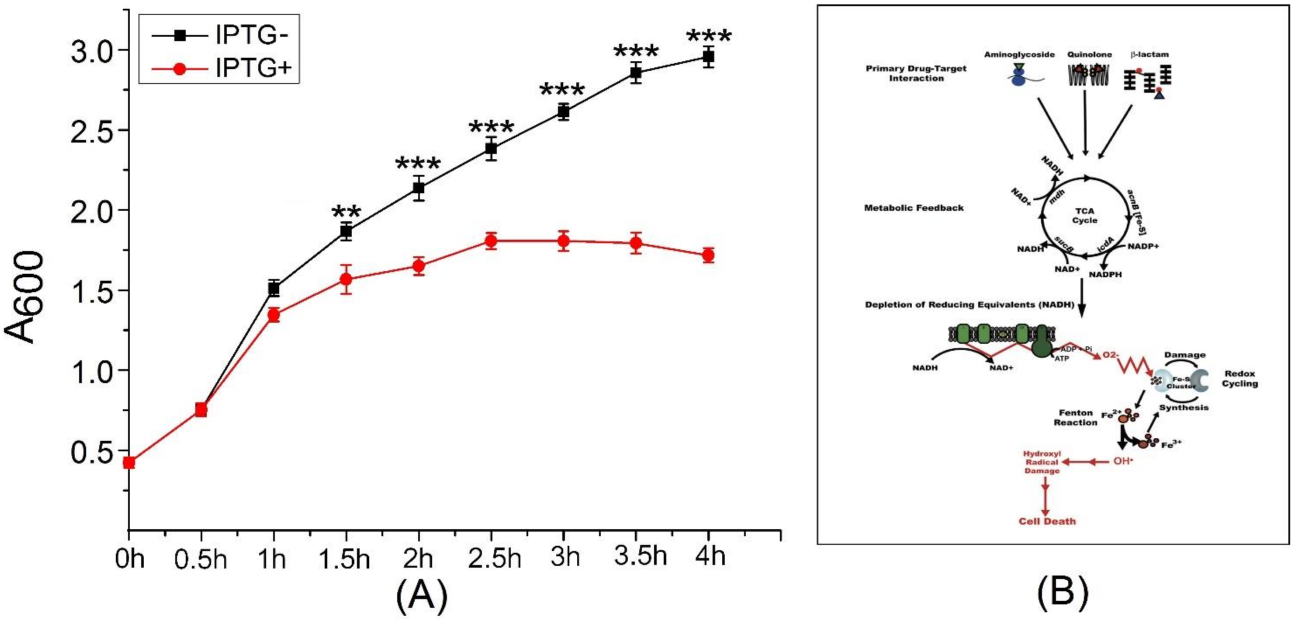

Supplementary Figure 5. (A) Growth curve of *E. coli* BL (DE3) induced or not by IPTG (Student's *t*-test, ***P < 0.001 and **P < 0.01). (B) Mechanism by which antibiotics promote autolysis of bacteria.

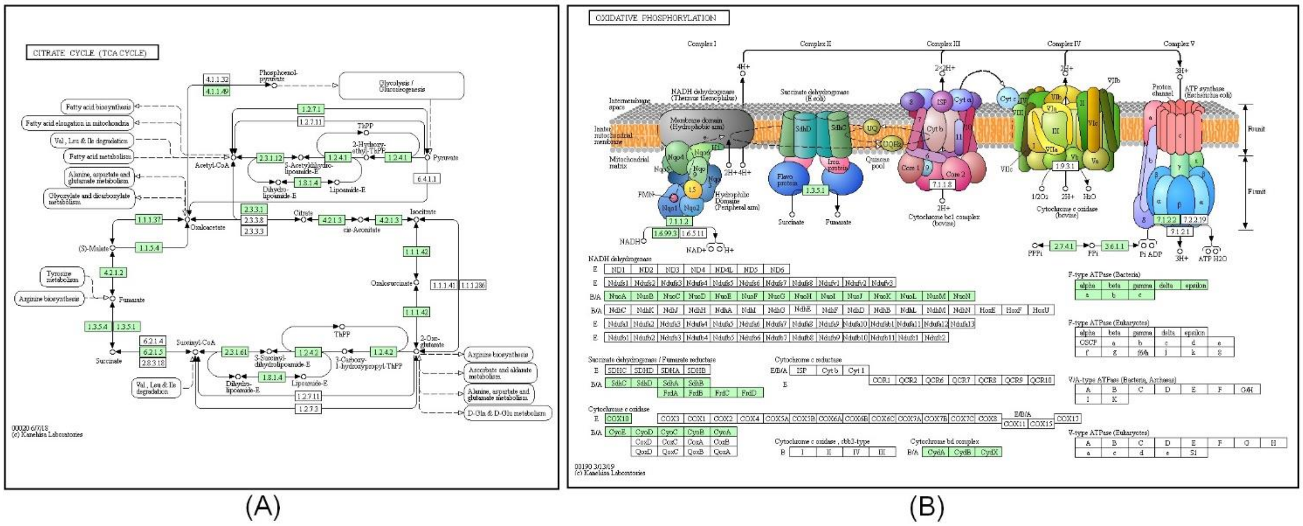

Supplementary Figure 6. (A) Kyoto Encyclopedia of Genes and Genomes (KEGG) pathway annotation of differentially expressed genes (DEGs) related to the TCA cycle; *acnA*, *ydbK*, *frdC*, *frdD*, *frdA*, and *frdB* were downregulated and *fumC*, *sdhD*, *sdhC*, *gltA*, *sdhB*, *sdhA*, and *sucA* were upregulated. (B) KEGG pathway annotation of DEGs related to oxidative phosphorylation; *nuoF*, *nuoE*, *nuoC*, *ppa*, *appC*, *frdC*, *appB*, *frdD*, *frdA*, and *frdB* were downregulated and *cyoA*, *cyoE*, *sdhD*, *sdhC*, *sdhB*, and *sdhA* were upregulated.

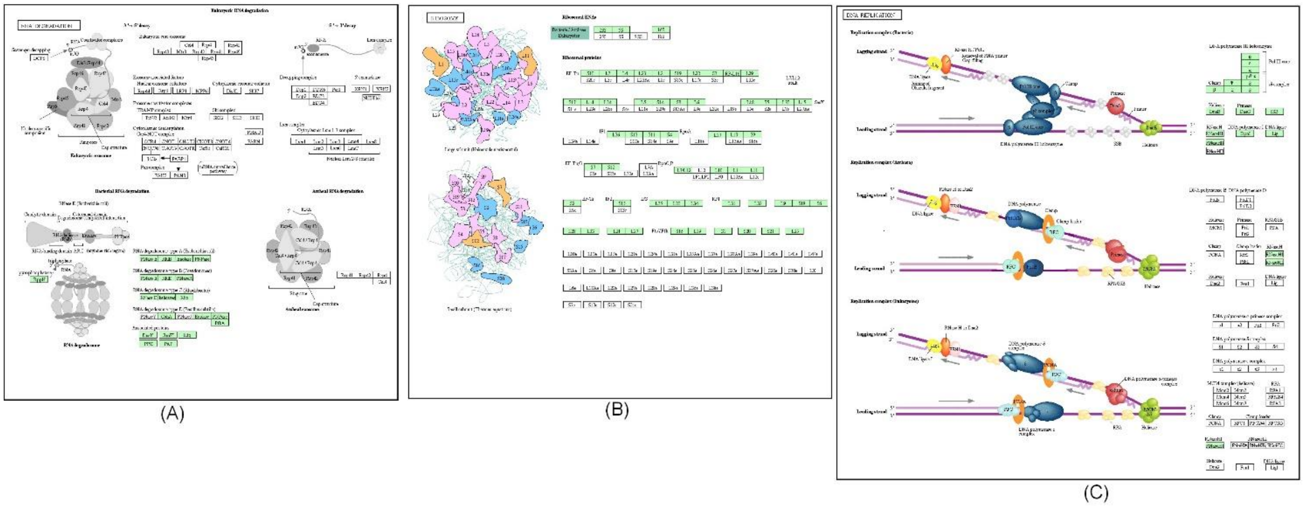

Supplementary Figure 7. (A) KEGG pathway annotation of DEGs related to RNA degradation; *groL*, *eno*, and *rne* were downregulated and *rhlB*, *hfq*, *rho*, and *rppH* were upregulated. (B) KEGG pathway annotation of DEGs related to the ribosome; *rplC*, *rplD*, *rplW*, *rplB*, *rpsS*, *rplV*, *rpsC*, *rplP*, *rpsM*, *rpsK*, *rplY*, *rplF*, *rpsD*, *rpsB*, *rpsJ*, *rplX*, *rplM*, *rpsH*, *rpsN*, *rpsE*, *rplR*, *rplK*, *rplA*, *rpsF*, *rpsG*, and *rpsL* were downregulated. (C) KEGG pathway annotation of DEGs related to DNA replication; *dnaE* was downregulated and *rnhA* was upregulated.

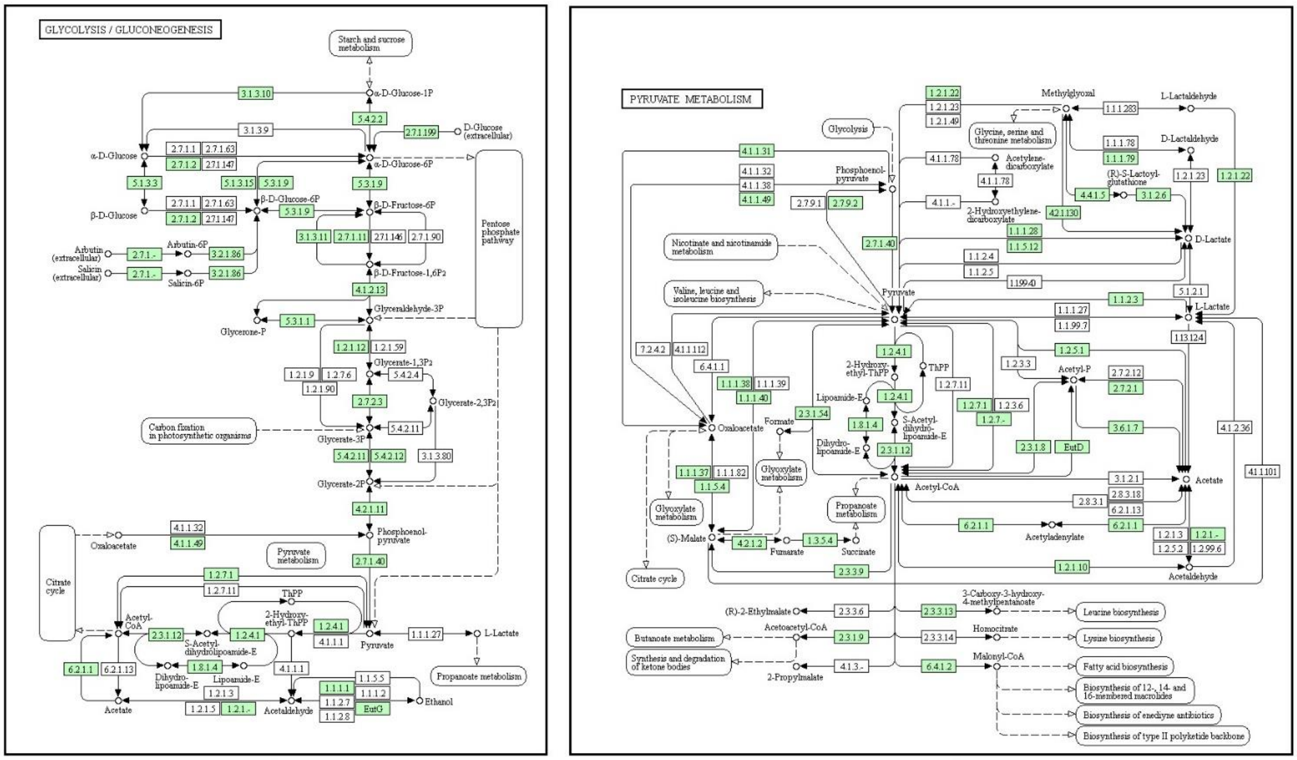

(A) (B)

Supplementary Figure 8. (A) KEGG pathway annotation of DEGs related to glycolysis/gluconeogenesis; *nuoF*, *nuoE*, *nuoC*, *ppa*, *appC*, *frdC*, *appB*, *frdD*, *frdA*, and *frdB* were downregulated. (B) KEGG pathway annotation of DEGs related to pyruvate metabolism; *poxB*, *NA*, *pykF*, *aldB*, *pykA*, *ydbK*, *frdC*, *frdD*, *frdA*, *frdB*, and *pflB* were downregulated.

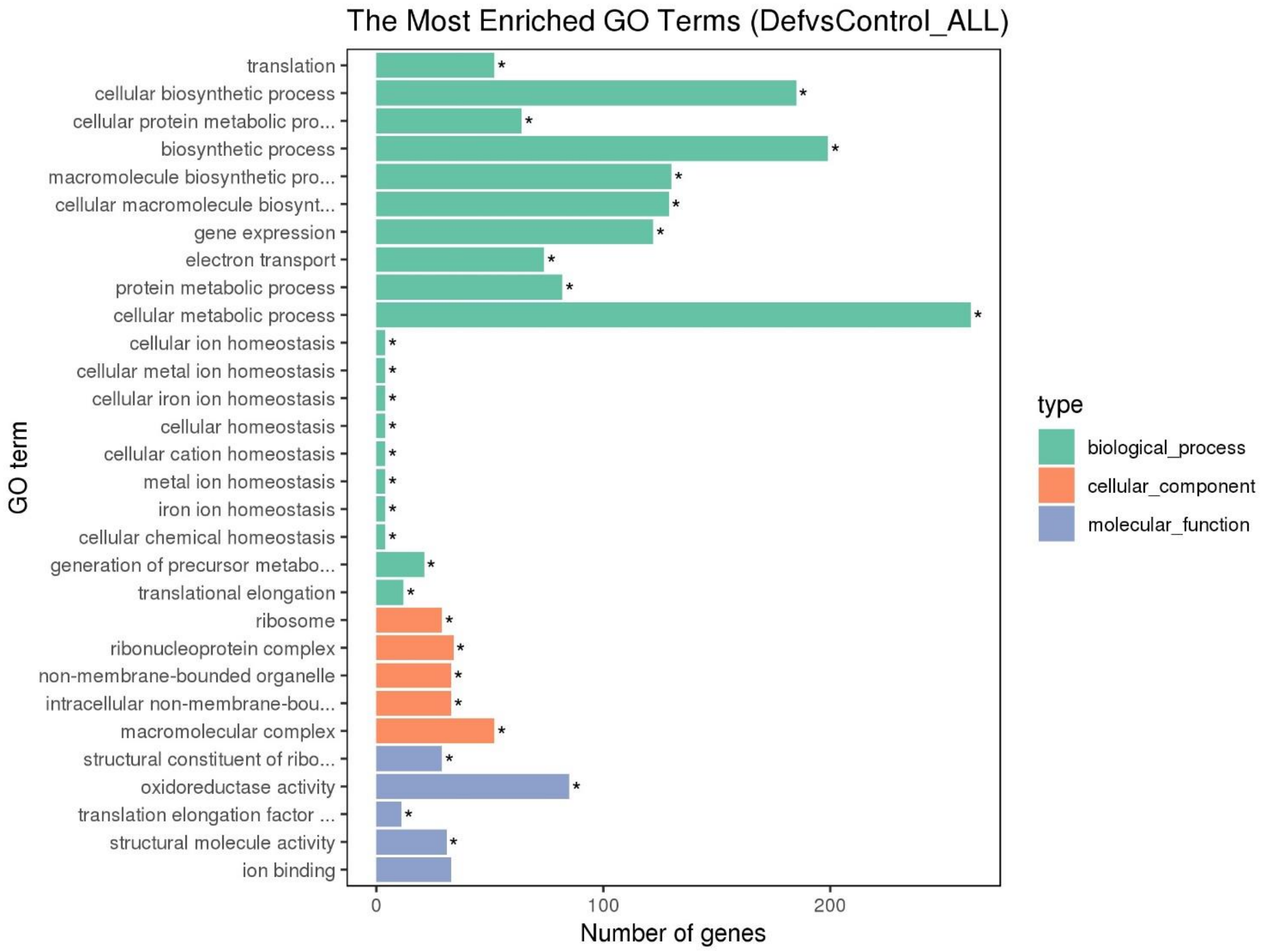

Supplementary Figure 9. KEGG pathway annotations of DEGs.

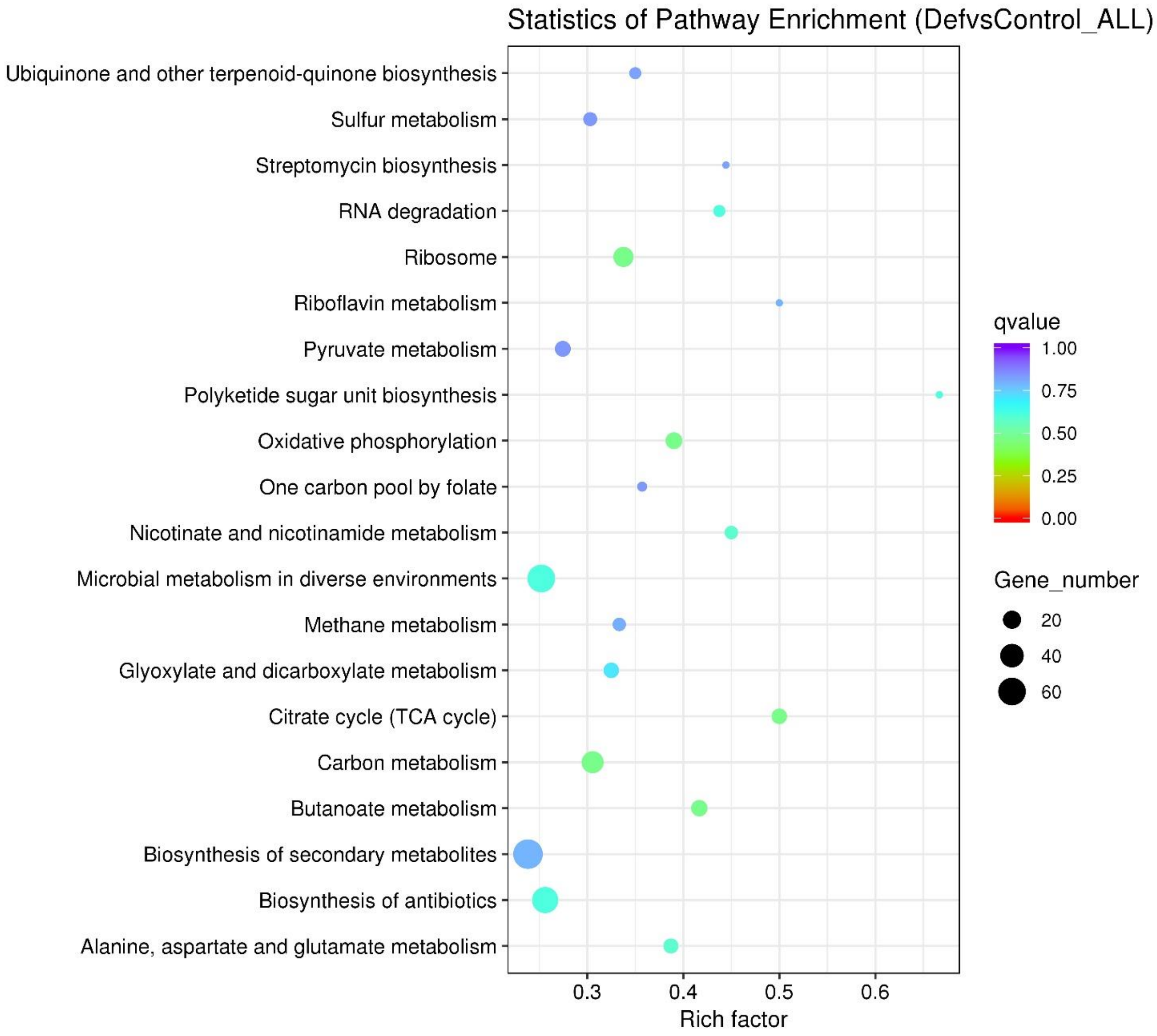

Supplementary **Figure 10.** Scatter plot of KEGG pathway enrichment. The abscissa is the enrichment factor of the pathway, the ordinate is the name of the pathway, the number of different genes in the pathway is indicated by the size of the point, and the size of the P-value is indicated by the color of the point (the smaller P-value, the closer the color to red).

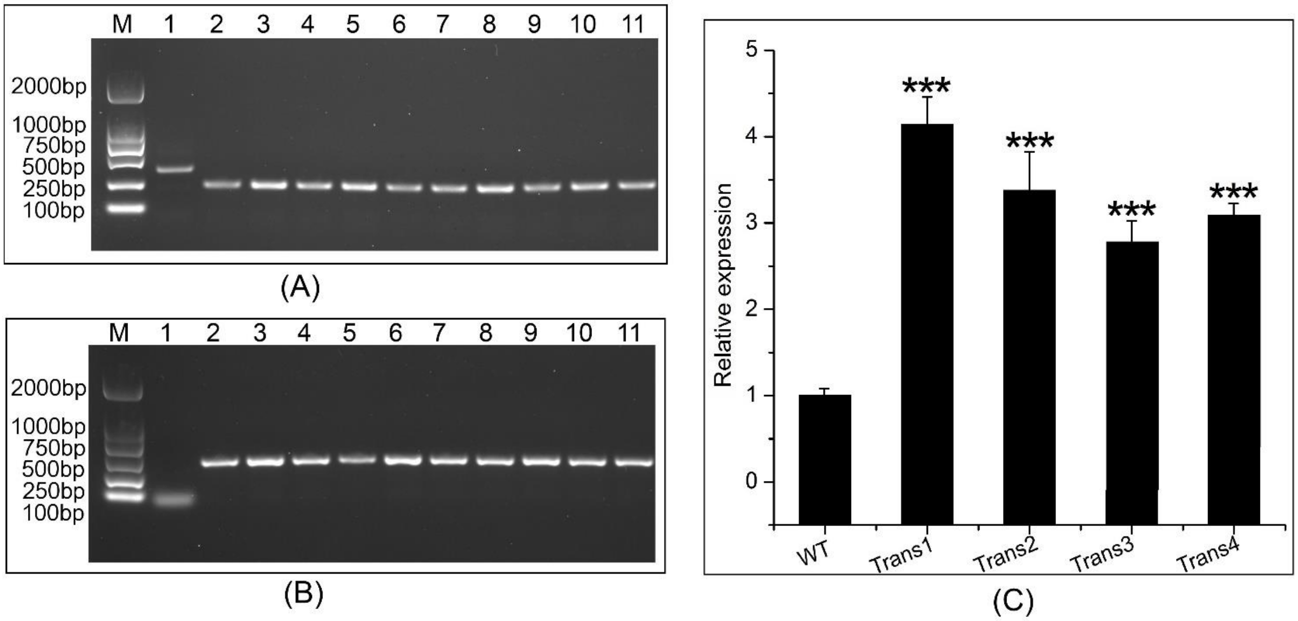

Supplementary Figure 11. Molecular identification of regenerated *PtDef* transgenic plants. (A) Identification of *PtDef* in the genome of transgenic and WT poplar by PCR using the PtDef-F and PtDef-R forward and reverse primers, respectively. Lane M, molecular mass marker; Lane 1, negative control (genome of WT poplar as the template); Lanes 2–11, transgenic lines 1–10 (Trans1–Trans10). (B) Identification of *PtDef* in the genome of transgenic and WT poplar by PCR using the 35S promoter as the forward primer and PtDef-R as the reverse primer. Lane M, molecular mass marker; Lane 1, negative control (WT poplar genome as the template); Lanes 2–11, transgenic lines 1–10 (Trans1–Trans10). (C) Analysis of *PtDef* expression in transgenic and WT poplar by real-time RT-PCR. Values are means ± SD of three biological replicates. Student's *t*-test, ***P < 0.001 compared to WT poplar.
